# Effect of Root Moisture Content and Diameter on Root Tensile Properties

**DOI:** 10.1371/journal.pone.0151791

**Published:** 2016-03-22

**Authors:** Yuanjun Yang, Lihua Chen, Ning Li, Qiufen Zhang

**Affiliations:** Key Laboratory of Soil and Water Conservation & Desertification Combat of Ministry of Education, School of Water and Soil Conservation, Beijing Forest University, Beijing, China; Tennessee State University, UNITED STATES

## Abstract

The stabilization of slopes by vegetation has been a topical issue for many years. Root mechanical characteristics significantly influence soil reinforcement; therefore it is necessary to research into the indicators of root tensile properties. In this study, we explored the influence of root moisture content on tensile resistance and strength with different root diameters and for different tree species. *Betula platyphylla*, *Quercus mongolica*, *Pinus tabulaeformis*, and *Larix gmelinii*, the most popular tree species used for slope stabilization in the rocky mountainous areas of northern China, were used in this study. A tensile test was conducted after root samples were grouped by diameter and moisture content. The results showedthat:1) root moisture content had a significant influence on tensile properties; 2) slightly loss of root moisture content could enhance tensile strength, but too much loss of water resulted in weaker capacity for root elongation, and consequently reduced tensile strength; 3) root diameter had a strong positive correlation with tensile resistance; and4) the roots of *Betula platyphylla* had the best tensile properties when both diameter and moisture content being controlled. These findings improve our understanding of root tensile properties with root size and moisture, and could be useful for slope stabilization using vegetation.

## 1. Introduction

Slope stabilization by vegetation is an environment-friendly, cost-effective, and sustainable engineering measure. The main factors contributing to slope stability include soil shear strength, soil-root interactions, the quantity and distribution of roots, as well as root tensile properties[1–4].Woody plants can reinforce soil by intertwining and exerting traction on the soil mass (in the case of lateral and fine roots) and by penetrating and anchoring into the ground (in the case of vertical and thick roots) [5–7]. Roots can be elongated due to tension deformation in response to soil shear force, and root tensile strength is therefore an essential indicator of root ability to resist external forces[8].

Over the past decades, many studies have demonstrated that root traits have a significant influence on root tensile properties, as do species, site condition, seasons of year, planting mode, and experimental displacement rate[4, 9–11]. Root traits include diameter, length, sinuosity, decay rate, orientation, and topology; they play an important role in hydrologic, mechanical, and ecological aspects of slope development [12–14]. Root diameter strongly affects tensile properties. Specifically, root diameter is negatively correlated with tensile strength and positively correlated with tensile resistance[1, 15–17].Root gauge length is another important factor to consider when measuring root strength. Some studies have shown that root tensile strength decreases with a linear increase in length [16, 18–19]. Cofie et al.(2001) found that root tensile strength can increase 8–20% with an increase in loading rate from 10 mm/m into 400 mm/min[20]. However, Zhang et al. (2012) did not observe significant differences in tensile strength at these two displacement rates[16]. In addition to the above, the effect of root chemical components on tensile properties has recently become a topical subject for researches. It was also found that the cellulose and lignin content in roots strongly affects tensile strength—cellulose and lignin can improve root elongation and hardness, respectively. With an increase in diameter, the percentage of cellulose increases while that of lignin decreases, with consequent decline in tensile strength[15, 21–22].

Osmanand Barakbah(2006) found that a slope with dense vegetation that has lower soil water content due to higher water absorption by roots beneath the ground exhibits better shear strength[23].It has been known that the presence of roots can enhance soil reinforcement, and that soil shear strength is negatively correlated with soil water content[23–25]. However, the relationship between root moisture content and corresponding mechanical characteristics has not been studied sufficiently. It is important to understand the influence of water on slope stabilization, not merely from the perspective of soil shear strength, but also with regard to root tensile strength. The aim of this study is to explore the influence of root moisture content on single root tensile properties, taking into consideration different root diameters and tree species. Moreover, through the quantitative and qualitative analysis on the relationship of tensile properties with root moisture content and root diameter, we investigated the effects of root moisture content and diameter on tensile properties.

## 2. Materials and Methods

### 2.1 Ethics statement

The sampling site is managed by Wudaohe Linchang, a state-owned forest Authority of He-bei province, China. Our study complies with the current laws of China and international rules. All necessary permits have been obtained before the actual sampling. The field study does not involve any endangered or protected species

### 2.2 Study area

Wudaohe Linchang(41°02'–42°14' N, 118°11'–118°29' E) covers an area of 7108.4ha. lying in the warm temperate zone with a continental monsoon climate. The temperature range is -26^−^36°C, and annual precipitation is 550 mm. Soil type is primarily loam and sandy loam brown earth. The vegetation is mainly warm temperate deciduous broad-leaved forest[26].

### 2.3 Sample collection and testing

Four tree species that are most common in our study area were selected; they are *Betula platyphylla*, *Quercus mongolica*, *Pinus tabulaeformis*, and *Larix gmelinii*. The first two species are broad-leaved species, while the latter two are conifers. We collected straight single roots to carry out tensile tests. Samples were collected twice, with the first samples used for pre-tests and the second for formal tests. The pre-tests were conducted to check the root moisture content range (the initial and air-dried moisture content) of each species. In the pre-tests, 20 samples of each species with different diameters (from1 to 7 mm) were weighed until the mass of each single root was constant under conditions of natural ventilation and temperature (25°C). The root initial moisture content and air-dry moisture content of each species were determined by the average value of 20 samples. Based on the results of pre-tests, the root moisture content ranges from the initial to air-dried condition are different between the four tested species due to their different roots characteristics. It is better to unify the classification number of each species in order to facilitate the next tests and analysis. We therefore considered the four ranges of root moisture content together and identified that seven stages (*H1*–*H7*) were the most proper for every species (Table 1). Root diameter was determined by the average of measured diameters at the points of trisection on the single root. Species roots were then also classified by diameter (Table 2). Even though root diameter changes with moisture loss, we used the initial root diameter as our standard diameter in this study in order to focus on exploring the effect of root moisture content.

**Table 1:** Root Moisture Content Stages.

Moisture content stage	*H1*	*H2*	*H3*	*H4*	*H5*	*H6*	*H7*
*Betula platyphylla*	47.00–54.00	40.00–47.00	33.00–40.00	26.00–33.00	19.00–26.00	12.00–19.00	5.00–12.00
*Quercus mongolica*	44.50–51.10	37.90–44.50	31.30–37.90	24.70–31.30	18.10–24.70	11.50–18.10	4.90–11.50
*Pinus tabulaeformis*	53.00–61.00	45.00–53.00	37.00–45.00	29.00–37.00	21.00–29.00	13.00–21.00	5.00–13.00
*Larix gmelinii*	51.00–58.65	43.35–51.00	35.7–43.35	28.05–35.70	20.40–28.05	12.75–20.40	5.10–12.75

**Table 2:** Root Diameter Classes.

Class	*D1*	*D2*	*D3*	*D4*	*D5*	*D6*
Diameter (mm)	1≤*D*<2	2≤*D*<3	3≤*D*<4	4≤*D*<5	5≤*D*<6	6≤*D*<7

In order to ensure that root moisture content measurements reflected true conditions, all samples were weighed (*M1*) (initial weight) within one day after collection and were then sealed tightly in bags. We also tested tensile resistance and recorded the diameter of samples in Stage *H1* (initial moisture content) before sealing the samples. The estimated weight range of each root in stages *H2-H7* was calculated as follows:

$$H_{n}=\frac{M_{n}-M_{dry}}{M_{dry}},\;H_{1}=\frac{M_{1}-M_{dry}}{M_{dry}}$$

the above formulae can be written as:

$$M_{n}=(H_{n}+1)M_{dry}$$ (1)

$$\text{and}\;M_{dry}=\frac{M_{1}}{H_{1}+1}$$ (2)

substitution Eq 2 into Eq 1 gives:

$$M_{n}=\frac{(H_{n}+1)M_{1}}{H_{1}+1}$$ (3)

therefore,

$$M_{nmin}=\frac{(H_{nmin}+1)M_{1}}{H_{1min}+1}$$ (4)

$$M_{nmax}=\frac{(H_{nmax}+1)M_{1}}{H_{1max}+1}$$ (5)

where

*M*_*1*_ is the root initial weight;

*H*_*1*_ is the initial root moisture content;

*M*_*n*_ is the estimated weight of the root in Stage *H*_*n*_;

*M*_*nmax*_ and *M*_*nmin*_ are the maximum and minimum weights of roots in Stage *H*_*n*_, respectively;

*H*_*1max*_ and *H*_*1min*_ are the maximum and minimum moisture contents of roots in Stage *H*_*1*_, respectively;

*H*_*nmax*_ and *H*_*nmin*_ are the maximum and minimum moisture contents of roots in Stage *Hn*, respectively; and *n* = 1, 2, 3, 4, 5, 6, 7.

Roots were placed indoors under conditions of natural ventilation and temperature (25°C). Each single root was weighed from time to time, and we then tested tensile properties until reaching each root’s own estimated weight range.

We carried out tensile tests with a WDW-100E electro-universal tester (Time Shijin, China) equipped with microcomputer control. The ranges of test force and speed are 400–100 kN and 0.001–500 mm/min, respectively. The machine has full automatic shift and stepless speed regulation functions. Its test load- and displacement-measuring accuracy is ±0.5%.

All samples were tested at the pulling speed of 10 mm/min and with a 50 mm gauge, while each single root was around 150mm in length (due to 50mm at each end being inserted into the instrument). Roots that slipped out or that were pulled apart inside or very near to the fixing jaws were discarded. In total, there were 4620 single roots tested, with 56.39% of them were treated as valid samples (Table 3).

**Table 3:** Statistics of Valid Samples.

Species	Moisture content	Total samples	Valid samples	Valid percentage (%)
*Betula platyphylla*	*H1*	173	86	49.71%
	*H2*	163	83	50.92%
	*H3*	156	80	51.28%
	*H4*	138	76	55.07%
	*H5*	126	72	57.14%
	*H6*	113	71	62.83%
	*H7*	139	75	53.96%
	Sum	1008	543	53.87%
*Quercus mongolica*	*H1*	194	101	52.06%
	*H2*	177	100	56.50%
	*H3*	186	100	53.76%
	*H4*	144	93	64.58%
	*H5*	121	84	69.42%
	*H6*	118	89	75.42%
	*H7*	110	77	70.00%
	Sum	1050	644	61.33%
*Pinus tabulaeformis*	*H1*	248	149	60.08%
	*H2*	237	140	59.07%
	*H3*	229	148	64.63%
	*H4*	197	137	69.54%
	*H5*	180	133	73.89%
	*H6*	203	122	60.10%
	*H7*	218	125	57.34%
	Sum	1512	954	63.10%
*Larix gmelinii*	*H1*	179	89	49.72%
	*H2*	151	80	52.98%
	*H3*	155	79	50.97%
	*H4*	148	81	54.73%
	*H5*	123	76	61.79%
	*H6*	136	77	56.62%
	*H7*	158	82	51.90%
	Sum	1050	564	53.71%
Total		4620	2705	58.55%

### 2.4 Data analysis tools

IBM SPSS Statistics version 22.0 was used for data analysis. Specifically, 1) outliers of observed values were rejected using the boxplot method; 2) data were tested for normality (using one-sample Kolmogorov-Smirnov) and variance homogeneity; 3) one-way analysis of variance (ANOVA) was used to test the significance of the effect of root diameter on tensile properties in each stage of root moisture content, and the effect of root moisture content on tensile strength in each diameter class; 4) analysis of covariance (ANCOVA) was used to test the significance of the effect of root moisture content on tensile resistance with root diameter classes as the covariate, moisture content stages as the independent variable, and root tensile resistance as the dependent variable; 5) regression analysis was used to quantify the relationship between root diameter and tensile resistance, and between moisture content stage and tensile strength.

## 3. Results

### 3.1 Effect of root diameter

ANOVA analysis of the effect of root diameter on root tensile properties was conducted under the conditions of controlled root moisture content (*H*). We took analysis of *Betula platyphylla* Group *H1*and *H3*asexamples. We concluded that the effect of root diameter on tensile properties was significant in the case of tensile resistance (*F*_*(5*, *80)*_ = 127.02, *P*<0.01 in *H1*; *F*_*(5*, *74)*_ = 97.16, *P*<0.01 in *H3*),but was unstable in the case of tensile strength- minimal in *H1*(*F*_*(5*, *80)*_ = 0.53, *P* = 0.75) and significant in *H3*(*F*_*(5*, *74)*_ = 4.23, *P*<0.01).Tests of other groups demonstrated similar result: there was significant difference in tensile resistance but inconclusive significance in tensile strength. Please see S2 File for ANOVA analysis of tensile properties affected by root diameter in each moisture content stage for the four species.

Regression analysis was used to explore the quantitative relationship between root diameter and tensile resistance. Power function was established taking into consideration of T-test and F-test values. A high *R* squared value indicated that the power function can predict root tensile resistance well on the basis of root diameter for all root moisture content stages; however, the model fitting degrees for *H6* and *H7* were relatively low compared to those for high water groups (Table 4). In addition, both our observations and models showed that root diameter had a significantly positive influence on single root tensile resistance.

**Table 4:** Summary of Regression Equations between Root Diameter and Tensile Resistance for Different Root Moisture Content Stages. *F*: Tensile resistance/kN. *D*: Diameter/mm.

Species	Moisture Content	Model	*R* Square	*P* Value
*Betula platyphylla*	*H1*	*F* = 0.018*D*^1.941^	0.92	<0.01
	*H2*	*F* = 0.019*D*^1.958^	0.92	<0.01
	*H3*	*F* = 0.025*D*^1.817^	0.94	<0.01
	*H4*	*F* = 00.024*D*^1.972^	0.95	<0.01
	*H5*	*F* = 0.029*D*^1.712^	0.88	<0.01
	*H6*	*F* = 0.038*D*^1.308^	0.75	<0.01
	*H7*	*F* = 0.021*D*^1.562^	0.72	<0.01
*Quercus mongolica*	*H1*	*F* = 0.018*D*^1.751^	0.89	<0.01
	*H2*	*F* = 0.019*D*^1.812^	0.90	<0.01
	*H3*	*F* = 0.021*D*^1.773^	0.92	<0.01
	*H4*	*F* = 0.023*D*^1.781^	0.88	<0.01
	*H5*	*F* = 0.022*D*^1.665^	0.80	<0.01
	*H6*	*F* = 0.019*D*^1.679^	0.76	<0.01
	*H7*	*F* = 0.023*D*^1.425^	0.68	<0.01
*Pinustabulaeformis*	*H1*	*F* = 0.009*D*^1.861^	0.88	<0.01
	*H2*	*F* = 0.009*D*^1.914^	0.87	<0.01
	*H3*	*F* = 0.009*D*^1.932^	0.87	<0.01
	*H4*	*F* = 0.007*D*^2.008^	0.81	<0.01
	*H5*	*F* = 0.005*D*^1.965^	0.71	<0.01
	*H6*	*F* = 0.004*D*^1.755^	0.61	<0.01
	*H7*	*F* = 0.003*D*^1.786^	0.66	<0.01
*Larix gmelinii*	*H1*	*F* = 0.009*D*^1.915^	0.86	<0.01
	*H2*	*F* = 0.011*D*^1.872^	0.90	<0.01
	*H3*	*F* = 0.010*D*^1.991^	0.90	<0.01
	*H4*	*F* = 0.010*D*^1.953^	0.93	<0.01
	*H5*	*F* = 0.011*D*^1.923^	0.86	<0.01
	*H6*	*F* = 0.011*D*^1.431^	0.57	<0.01
	*H7*	*F* = 0.009*D*^1.413^	0.45	<0.00

### 3.2 Effect of root moisture content

#### 3.2.1 Tensile properties

It has been confirmed that root diameter has significant influence on tensile resistance but minor on tensile strength, so we applied ANCOVA and ANOVA to explore the effect of root moisture content on tensile resistance and tensile strength, respectively. The ANCOVA results showed that the effect of root moisture content is significant for *Betula platyphylla*(*F*_*(6*, *536)*_ = 35.27, *P*<0.01 for moisture content; *F*_*(6*, *536)*_ = 67.59, *P*<0.01 for diameter class), *Quercus mongolica*(*F*_*(6*, *637)*_ = 12.21, *P*<0.01 for moisture content; *F*_*(6*, *637)*_ = 115.33, *P*<0.01 for diameter class) and *Pinus tabulaeformis (F*_*(6*, *947)*_ = 122.86, *P*<0.01 for moisture content; *F*_*(6*, *947)*_ = 8.90, *P*<0.01 for diameter class); but not for *Larix gmelinii*(*F*_*(6*, *557)*_ = 0.72, *P* = 0.64 for moisture content; *F*_*(6*, *557)*_ = 4.60, *P* = 0.03 for diameter class). In addition, ANOVA analysis of root tensile strength was conducted in each diameter class of different species, as affected by root moisture content. Results indicated that there is a significant difference in root tensile strength in different moisture content stages for the four tested species, except in *D1* class of *Quercus mongolica*, and in *D1* class and *D5*class of *Larix gmelinii* (Table 5).

**Table 5:** ANOVA Results of Relationship Between Root Tensile Strength and Moisture Content.

Species	Diameter class	*F* value	*P* value
*Betula platyphylla*	*D1*	8.30	<0.01**
	*D2*	12.53	<0.01**
	*D3*	7.46	<0.01**
	*D4*	8.61	<0.01**
	*D5*	15.04	<0.01**
	*D6*	15.53	<0.01**
*Quercus mongolica*	*D1*	1.20	0.32
	*D2*	4.14	<0.01**
	*D3*	5.72	<0.01**
	*D4*	4.84	<0.01**
	*D5*	1.03	<0.41
	*D6*	5.72	<0.01**
*Pinus tabulaeformis*	*D1*	23.75	<0.01**
	*D2*	45.81	<0.01**
	*D3*	37.00	<0.01**
	*D4*	19.72	<0.01**
	*D5*	16.37	<0.01**
	*D6*	14.58	<0.01**
*Larix gmelinii*	*D1*	0.39	0.88
	*D2*	17.30	<0.01**
	*D3*	16.03	<0.01**
	*D4*	10.86	<0.01**
	*D5*	12.42	<0.01**
	*D6*	9.18	<0.01**

**means the difference is significant at the 0.01 level

We conducted quantitatively analysis for the effect of root moisture content on tensile strength but not for the effect of that on tensile resistance; this was due to the fact that strength is an effective parameter for reflecting the mechanical characteristics of specimens, eliminating the influence of disturbance from size and shape[27–28]. We used regression analysis of *Betula platyphylla* as an example to explore the correlation of root moisture content and tensile strength. The quadratic curve equation is the best model to explain their quantitative relationship, even though variability was high (Table 6). Regression analysis of the three other experimental species revealed similar outcomes. All models were significant, indicating that the tensile strength was related to root moisture content. The lower fitting degree means that some other variables may also play an important role in tensile strength. Besides, the moisture content stage represents a change range but not the exact content data, which also could be part of the reason for the high variability of the regression of root tensile strength and moisture content stage.

**Table 6:** Regression Analysis of Root Moisture Content Stage and Tensile Strength for *Betula platyphylla*. *T*: Tensile strength/Mpa. *H*: Moisture content. *D*: Diameter class.

*D*	Model	*R* square	*P* value
1	*T* = -1.207*H*^*2*^+10.001*H*+11.127	0.40	<0.01
2	*T* = -1.162*H*^*2*^+9.257*H*+12.746	0.33	<0.01
3	*T* = -0.668*H*^*2*^+5.127*H*+17.435	0.13	<0.01
4	*T* = -1.038*H*^*2*^+7.513*H*+13.736	0.28	<0.01
5	*T* = -1.287*H*^*2*^+8.142*H*+14.750	0.49	<0.01
6	*T* = -0.933*H*^*2*^+6.108*H*+13.783	0.47	<0.01

**Fig 1:**
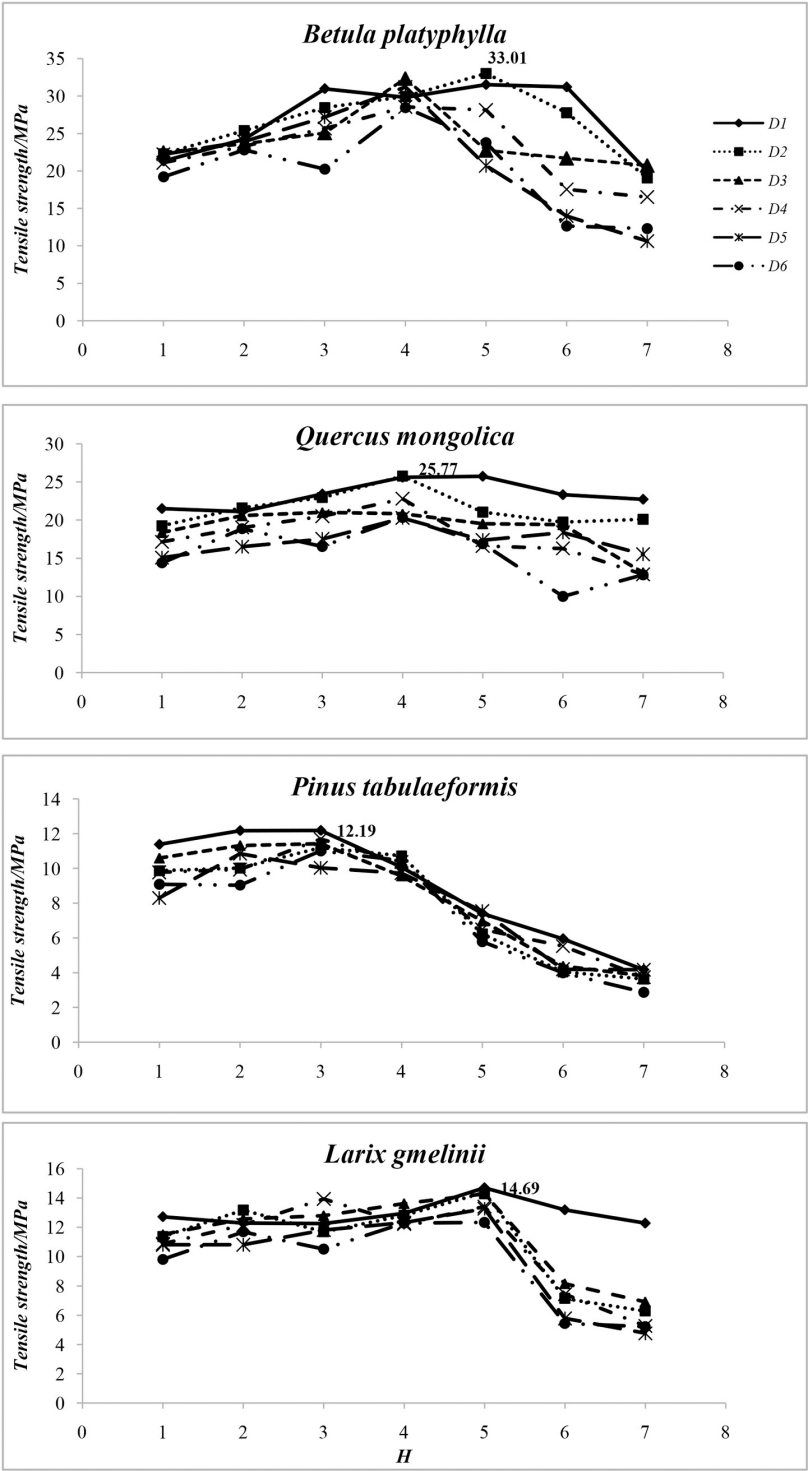
Trend Graphs of Root Tensile Strength with Moisture Content for All Diameter Classes. *H*: Root moisture content stage.

There was no model that fit very well to predict the quantitative relationship between root moisture and corresponding tensile strength, however, the qualitative relationship between them was worthy to explore. Trend graphs (Fig 1) were drawn based on average tensile strength of each moisture content stage. These graphs show that: 1) Lowest strength occurred in *H7* in all diameter groups for each experimental species. 2) Taking the different diameter classes into consideration, the best root tensile strength mostly appeared in *H4* for *Betula platyphylla* and *Quercus mongolica* (26.00–33.00% and 24.70–31.30%, respectively), in *H3* for *Pinus tabulaeformis* (37.00–45.00%), and in *H5* for *Larix gmelinii* (20.40–28.05%). 3) On the whole, the sequence from top to bottom of curves showed that root tensile strength decreased with diameter increasing. Nevertheless, this rule was not appropriate for every diameter class. 4) There was an evident turning point for two coniferous species (*H3* for *Pinus tabulaeformis* and *H5* for *Larix gmelinii*), tensile strength decreased sharply after the turning point. Curves of *Betula platyphylla* and *Quercus mongolica* were irregular. 5) The strongest tensile strengths of *Betula platyphylla*, *Quercus mongolica*, *Pinus tabulaeformis*, and *Larix gmelinii* were 33.01MPa (in *H5* with *D2*), 25.77MPa (in *H4* with *D2*), 12.19MPa (in *H3* with *D1*), and 14.69MPa (in *H5* with *D1*), respectively. 6) *Betula platyphylla* performed best in terms of single root tensile strength. Furthermore, the two deciduous species performed much better than the two conifers in terms of tensile strength. The order of tensile strength from high to low was *Betula platyphylla*, *Quercus mongolica*, *Larix gmelinii*, and *Pinus tabulaeformis*.

#### 3.2.2 Stress-strain curve

**Fig 2:**
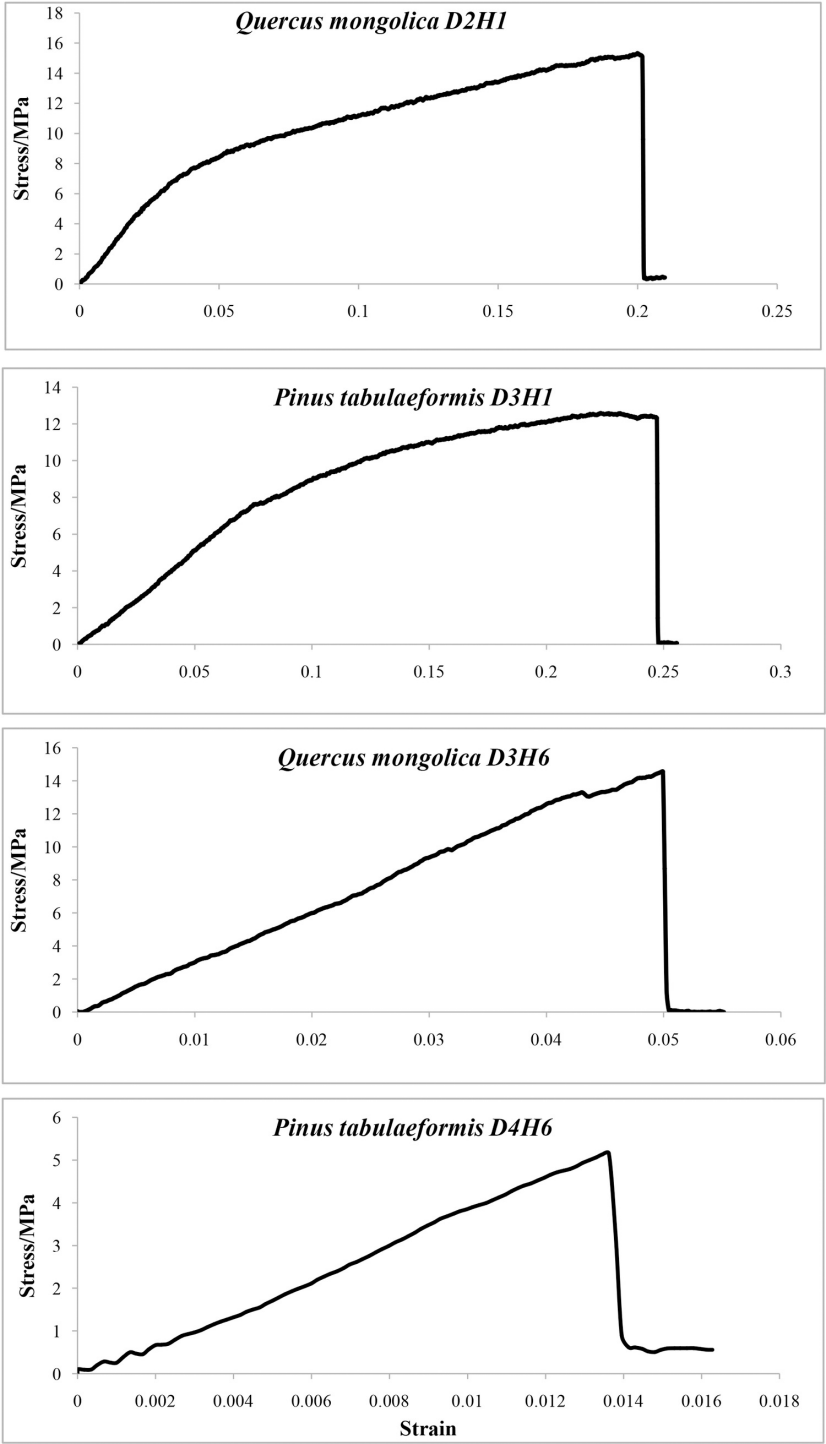
Four Examples of Stress-Strain Curves. *Quercus mongolica D2H1*: one *Quercus mongolica*sample with diameter in *D2* class and moisture content in *H1*stage; *Pinus tabulaeformis D3H1*: one *Pinus tabulaeformis* sample with diameter in *D3* class and moisture content in *H1* stage; *Quercus mongolica D3H6*: one *Quercus mongolica* sample with diameter in *D3* class and moisture content in *H6*stage; *Pinus tabulaeformis D4H6*: one *Pinus tabulaeformis* sample with diameter in *D4* class and moisture content in *H6*stage.

The influence of root moisture content on tensile strength can also be presented through a stress-strain curve. Four examples are given in Fig 2. The first two were for moist roots and the last two were for dry roots. The curves of moist roots illustrated that: 1) in the early stage of loading, stress increased linearly with strain increasing, roots showed linear elasticity; 2) subsequently, stress surpassed the elastic limit and its increase slowed, roots began to be subject to elastic plastic deformation; 3) the curve slope became smaller and stress increased more and more slowly, until root breakage occurred. In contrast, the curves of dry roots only showed linear deformation before breakage.

#### 3.2.3 Maximum improvement rate of root tensile strength

The maximum improvement rate of root tensile strength enhanced by the change of moisture content compared with that in the initial moisture content was summarized in Table 7. A certain loss of root moisture content could improve tensile strength in the range of 4.59–48.18%. Among the four experimental species, tensile strength improved most significantly and steadily in the case of *Betula platyphylla*. Moreover, the broadleaves acquired higher improvement in root tensile strength than the conifers.

**Table 7:** The Maximum Improvement Rate of Root Tensile Strength. *D*: Diameter class. *H1*: Initial root moisture content. SD: Standard deviation.

Species	*D*	Strength of *H1* (mean±SD/MPa)	Maximum strength (mean±SD/MPa)	Improvement rate (%)
*Betula platyphylla*	1	21.32±5.60	30.99±5.23	45.39%
	2	22.28±6.45	33.01±5.65	48.18%
	3	22.49±4.79	32.35±5.97	43.84%
	4	21.09±6.56	28.56±6.16	35.40%
	5	22.20±5.45	31.21±6.14	40.55%
	6	19.22±3.67	28.46±3.30	48.07%
*Quercus mongolica*	1	21.51±5.40	25.72±5.96	19.57%
	2	19.19±4.62	25.77±6.49	34.27%
	3	18.36±5.02	20.95±4.37	14.11%
	4	17.13±5.01	22.78±5.49	32.97%
	5	15.09±3.03	20.26±5.51	34.22%
	6	14.60±2.05	20.34±5.72	39.29%
*Pinus tabulaeformis*	1	11.38±2.80	11.91±3.25	4.59%
	2	9.87±3.41	11.19±3.49	13.39%
	3	10.92±3.43	11.43±3.93	4.62%
	4	9.78±2.97	11.70±3.00	19.61%
	5	8.30±2.86	10.87±2.28	30.90%
	6	9.09±2.11	11.02±2.90	21.23%
*Larix gmelinii*	1	12.71±3.72	14.69±2.51	15.53%
	2	11.37±3.19	14.38±3.41	26.53%
	3	11.50±2.92	14.28±3.36	24.17%
	4	10.88±2.95	13.41±2.92	28.12%
	5	10.80±2.04	13.26±2.59	22.79%
	6	9.81±1.99	12.34±2.05	25.79%

### 3.3 Combined effect of root diameter and moisture content

**Fig 3:**
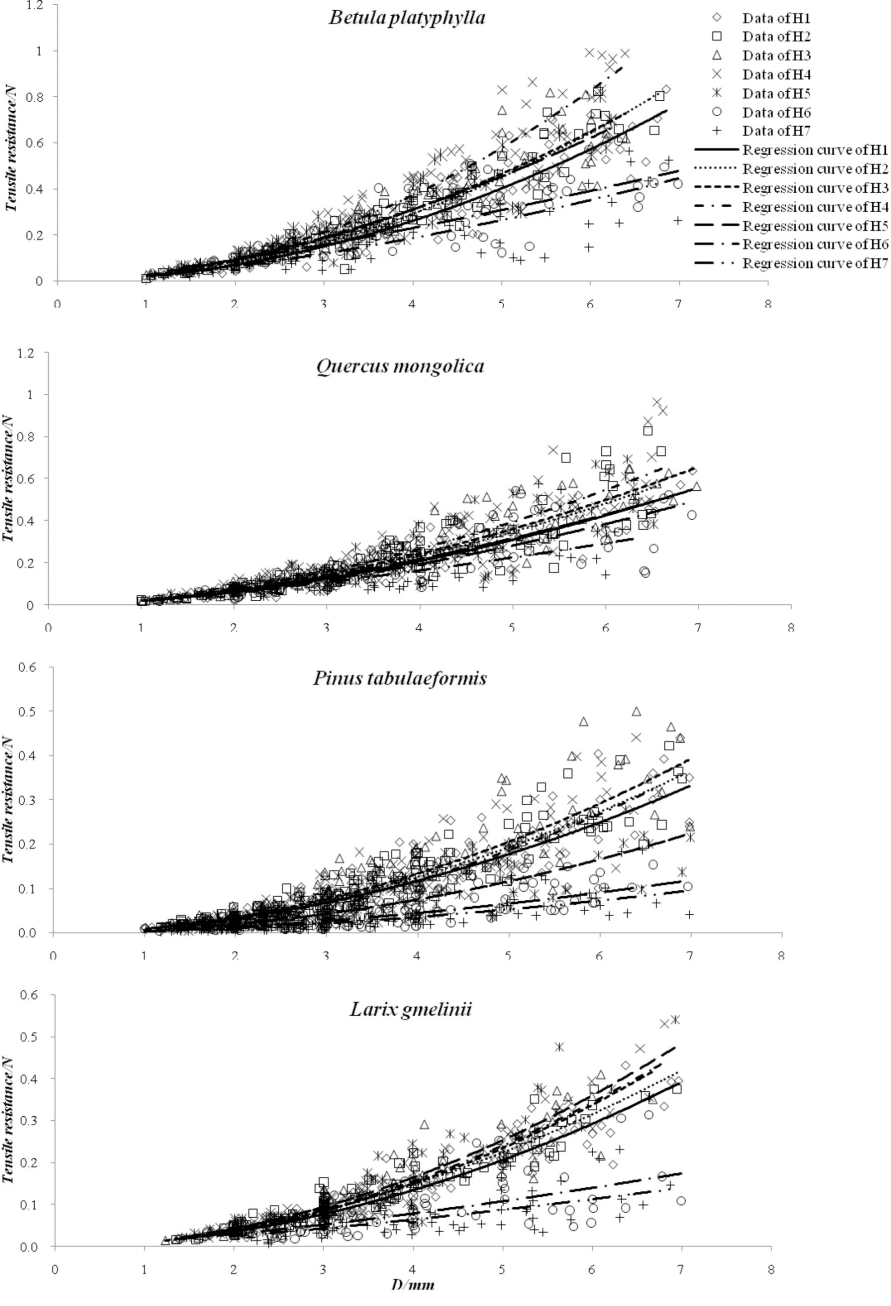
Model Curves of Root Tensile Resistance with Root Diameter for All Moisture Content Stages. *D*: Diameter/mm.

In order to compare model curves for all root moisture content stages (Fig 3), the influence of root diameter and moisture content was comprehensively analyzed: 1) Root tensile resistance grew in power function with the increase of diameter, which was listed in Table 4. 2) The curve slope was an indicator of the effect of root diameter on tensile resistance. In the case of all four species, tensile resistance in *H7* increased most slowly with root diameter; meanwhile, the effect was greatest in *H4* for *Betula platyphylla* and *Quercus mongolica*, in *H3* for *Pinus tabulaeformis*, and in *H5* for *Larix gmelinii*. This also indicated that the lowest and second lowest tensile resistance occurred in *H7* and *H6* respectively for the four species, and the maximum occurred in *H4* for *Betula platyphylla* and *Quercus mongolica*, in *H3* for *Pinus tabulaeformis*, and in *H5* for *Larix gmelinii*. 3) When both root diameter and moisture content were controlled, *Betula platyphylla* generally possessed the best tensile resistance, followed by *Quercus mongolica*, *Larix gmelinii*, and *Pinus tabulaeformis*.

## 4. Discussion

### 4.1 Root tensile resistance and strength

Our results showing a positive power function correlation between root diameter and tensile resistance is comparable to the results from most previous studies[15–17, 29]. The influence of root diameter on tensile properties could be explained from the perspective of chemical components. Cellulose percentage increases, lignin percentage decreases and therefore lignin-cellulose ratio decreases as root diameter increases. Since lignin-cellulose ratio is significantly positively correlated with tensile strength, thus, tensile strength decreases with increasing root diameter [1, 15, 21, 30]. Previous studies have confirmed a negative correlation between root diameter and tensile strength, following a power function[1, 4, 15, 17, 31–33]. We also observed a decrease in root tensile strength with an increased diameter in this study; nevertheless, the influence of root diameter on tensile strength was not always significant. This can be explained by the autocorrelation between tensile strength and diameter, to be more specific:

$$\sigma =\frac{F}{S}=\frac{4F}{\pi D^{2}}$$ (6)

Where *σ* is root tensile strength, *F* is root tensile resistance, *S* is root area, and *D* is root diameter.

Based on the modeled relationship between root tensile resistance and diameter (Table 4):

$$F=aD^{b}$$ (7)

Where *a* and *b* are constants.

Combining Eq 7 with Eq 6 gives:

$$\sigma =\frac{4aD^{b}}{\pi D^{2}}=\frac{4a}{\pi }D^{\left(b-2\right)}$$ (8)

Based on the regression equations between root diameter and tensile resistance (Table 4), 1<*b*<2 (except *H4*stage of *Pinus tabulaeformis*), then (*b*-2)<0, so root diameter is negatively correlated with tensile strength. However, it is not possible to find a fitting model to express this correlation. On the one hand, the power of (*b*-2) reduces the influence of root diameter on tensile strength, which directly results in the insignificance of ANOVA analysis of the effect of diameter on tensile strength. On the other hand, we can see from Eq 6 that root tensile strength is determined by tensile resistance and diameter, whereas tensile resistance is a dependent variable as well. Some errors may have occurred when modeling the relationship between root diameter and tensile resistance, so it is reasonable that a model to predict root tensile strength on the basis of root diameter cannot be easily deduced from Eq 8.

### 4.2 Stress-strain curves reflecting mechanical properties

The stress-strain curve is an important reflection of materials’ mechanical properties and can comprehensively illustrate the tensile process. A plant root is a type of natural elastic-plastic material[34]. Our curves (with linear and nonlinear deformation) (Fig 2) implied the elastoplasticity of moist roots. Dry roots could be viewed as a type of elastic material but its elasticity is weak. Based on these mechanical characteristics, moist roots are more conducive to soil reinforcement than dry roots. Specifically, moist roots can break beyond their elastic limit stress due to nonlinear deformation of elastoplasticity, while dry roots can only break at a certain stress limit; this means that the moisture root with greater elongation can perform better in spreading loading force to deeper soil, imparting resilience to the soil under shear force.

### 4. 3 Effect of root bark

Root bark has a significant influence on root tensile properties[33–34]. It was anticipated that root bark would have a more positive effect on root mechanical properties when roots hold the proper amount of water. This effect was potentially more evident for *Pinus tabulaeformis* and *Larix gmelinii*, since the barks of these species cracked and peeled off when they dried out. Additionally, deciduous species had better single root tensile properties than conifers. These differences could be partly due to root bark, as expected. To be more specific, compared with the thin and loose root bark of *Pinus tabulaeformis* and *Larix gmelinii*, roots of *Betula platyphylla* and *Quercus mongolica*were covered by much thicker and more intact root bark, with this playing an important role in protecting roots from chemical and physical damage. The root bark of *Betula platyphylla* and *Quercus mongolica* couldstay intact even when the root was extremely dry and could thus continue to serve a protective function. On the other hand, the root bark of the two conifers protected roots less effectively; when the bark was cracked, root tensile strength fell greatly- as the turning points were *H3* for *Pinus tabulaeformis* and *H5* for *Larix gmelinii* (Fig 1).

### 4.4 Success rate of tensile tests

We found that the highest valid percentage of tensile tests appeared in roots with moisture content *H5* for *Pinus tabulaeformis* and *Larix gmelinii*, and *H6* for *Betula platyphylla* and *Quercus mongolica*. One possible reason for this phenomenon is that roots with higher moisture content were prone to slip out from the jaws of the clamping devices during tests, while roots with lower moisture content always broke while being fixed on the instrument (before this started pulling).

### 4.5 Limitations and further study

Samples used for pre-tests were collected in July, 2014, while samples for formal tests were collected in October, 2014. Roots were found to have better tensile properties in winter than in summer, because of lower root moisture content in winter[35]. There might thus be some errors in terms of root initial moisture content, even though the same drying conditions were expected to give the same air-dried moisture content. Unavoidably, the moisture content range might not be very accurate.

In addition, diameter was measured with root bark in this study. However, features like thickness and hardness of bark differ for each species. The presence of root bark affects root diameter and tensile properties[34]. Lv(2012) found that the roots without bark have higher lignin-cellulose ratio than the roots with bark (when they have the same diameter), therefore roots without bark have better tensile strength[33]. Considering the significant effect of bark on tensile strength, we suggest that it is needed to further study on the mechanisms by which bark affected single-root tensile properties in future studies.

In this study, we focused mainly on the influence of root moisture content and diameter on single tensile properties. In reality, slope stabilization is determined by a number of factors. These include factors related to root tensile properties (such as genetic adaptation, root length, tensile loading speed, root moisture content, and root diameter) and factors related to root-soil shear strength (such as root distribution, root to soil volume, root biomass, elevation, sampling position on hillslope and soil water content) [23, 36–37]. The structural chemistry of roots is associated with soil water potential [30]. Besides, there is a close relationship between root moisture content and soil water content, and between soil shear strength and soil water content. It is important to further investigate the relationship among soil water content, root moisture content, and root tensile strength, coupled with root-soil shear strength.

## 5. Conclusion

In this paper we focused on analyzing single root tensile properties, as affected by root moisture content and diameter. The main findings of our study could be used as a reference for future studies, and are summarized below:

1. Root diameter had a strong effect on tensile resistance. Root tensile resistance increased with diameter; however, the effect of diameter on tensile strength was inconclusive significant.
2. Root moisture content significantly influenced tensile strength. The quadratic equation was the most applicable model for explaining the relationship of root moisture content and tensile strength; nonetheless, variability was high. Roots were weakest when extremely dry (e.g., moisture stages *H6* and *H7*). Roots with moderate moisture content had better tensile strength than those with initial and air-dried moisture content. The moisture content ranges with best tensile properties for *Betula platyphylla*, *Quercus mongolica*, *Pinus tabulaeformis*, and *Larix gmelinii* were 26.00–33.00%, 24.70–31.30%, 37.00–45.00%, and 20.40–35.70%, respectively. A certain loss of moisture (from initial moisture content) could improve tensile strength by 4.59–48.18%for the four experimental species, and the tensile strength of broadleaves was improved more than that of conifers.
3. There were significant differences in tensile properties between the four experimental species, with the order of strength of roots (from strong to weak) being *Betula platyphylla*, *Quercus mongolica*, *Larix gmelinii*, and *Pinus tabulaeformis*; the tensile properties of the two broad-leaved trees were apparently better than those of conifers.
4. Moist roots had elastoplasticity, while dry roots (*H6* and *H7*)only possessed very weak elasticity; the tensile strength of dry roots was thus much weaker than that of moist roots.

## Supporting Information

S1 File Experiment Data. (XLSX)

S2 File ANOVA Analysis of Tensile Resistance and TensileStrength Affected by Root Diameter. (XLSX)
